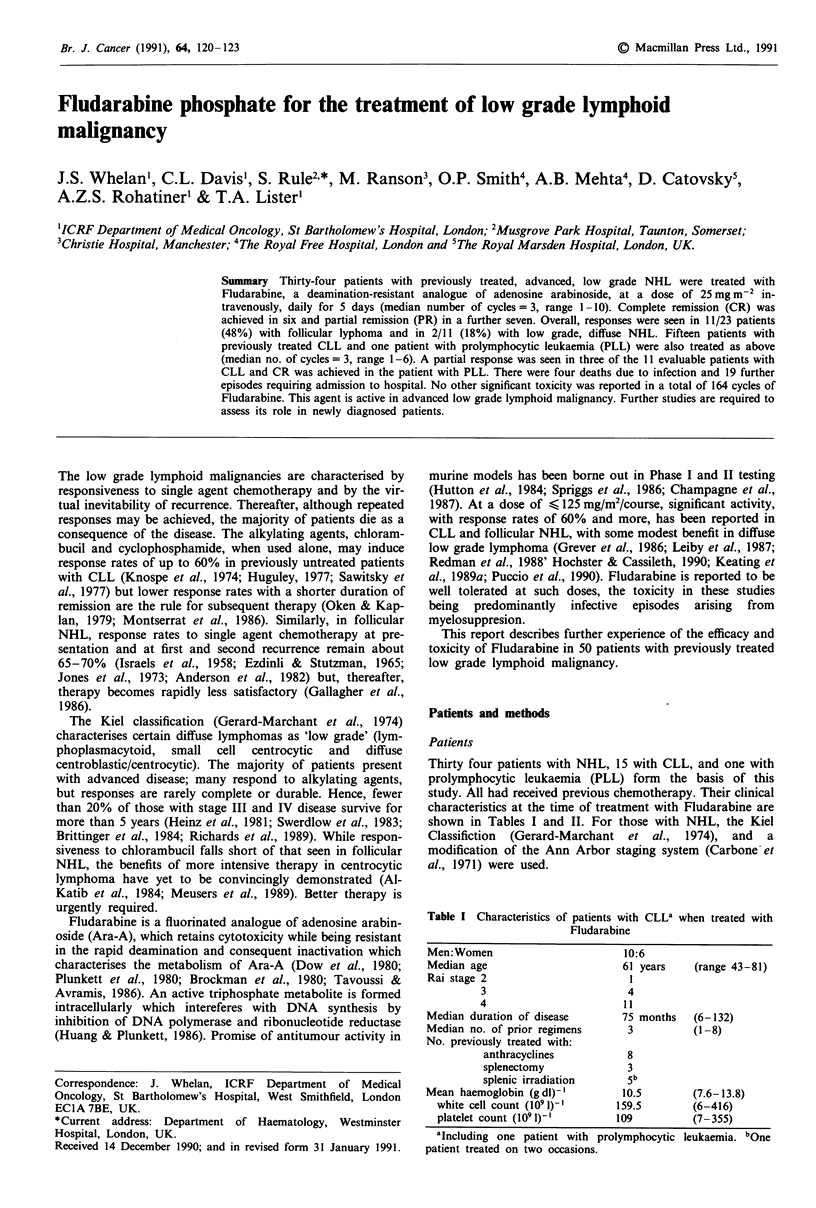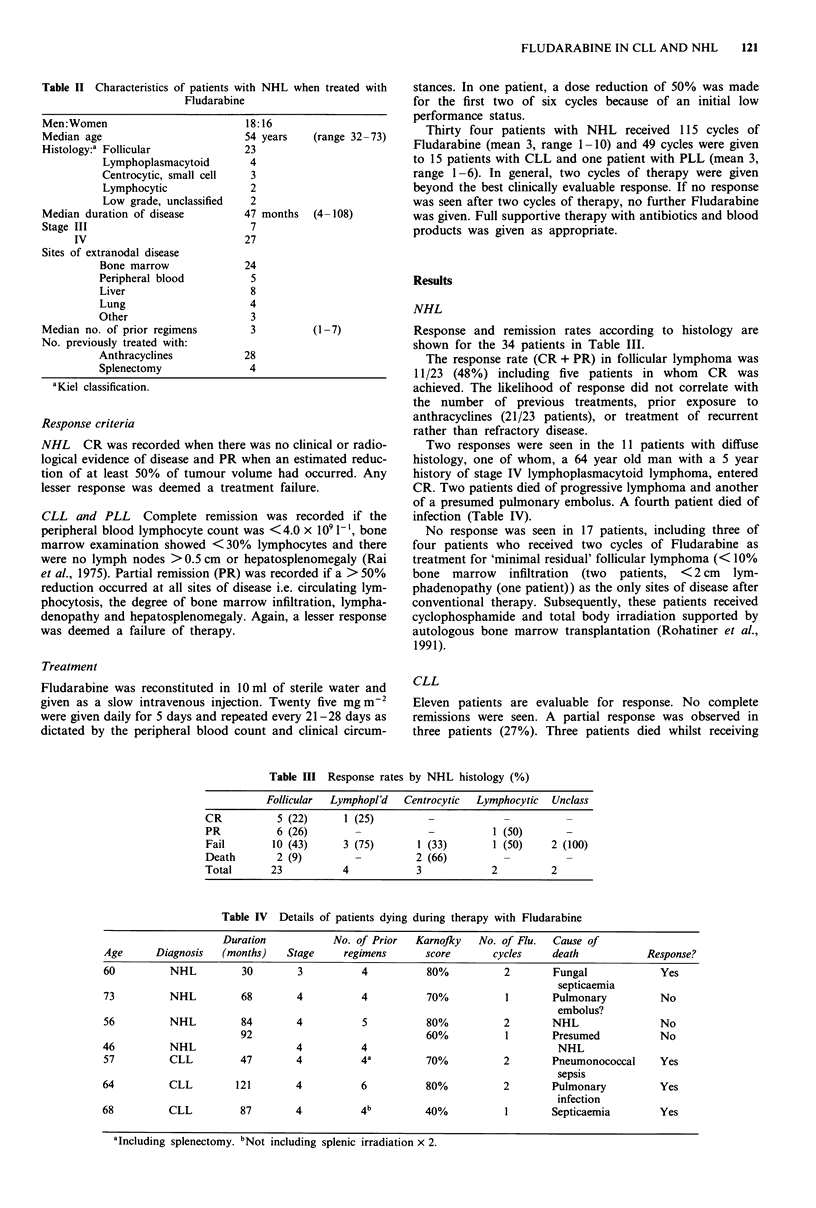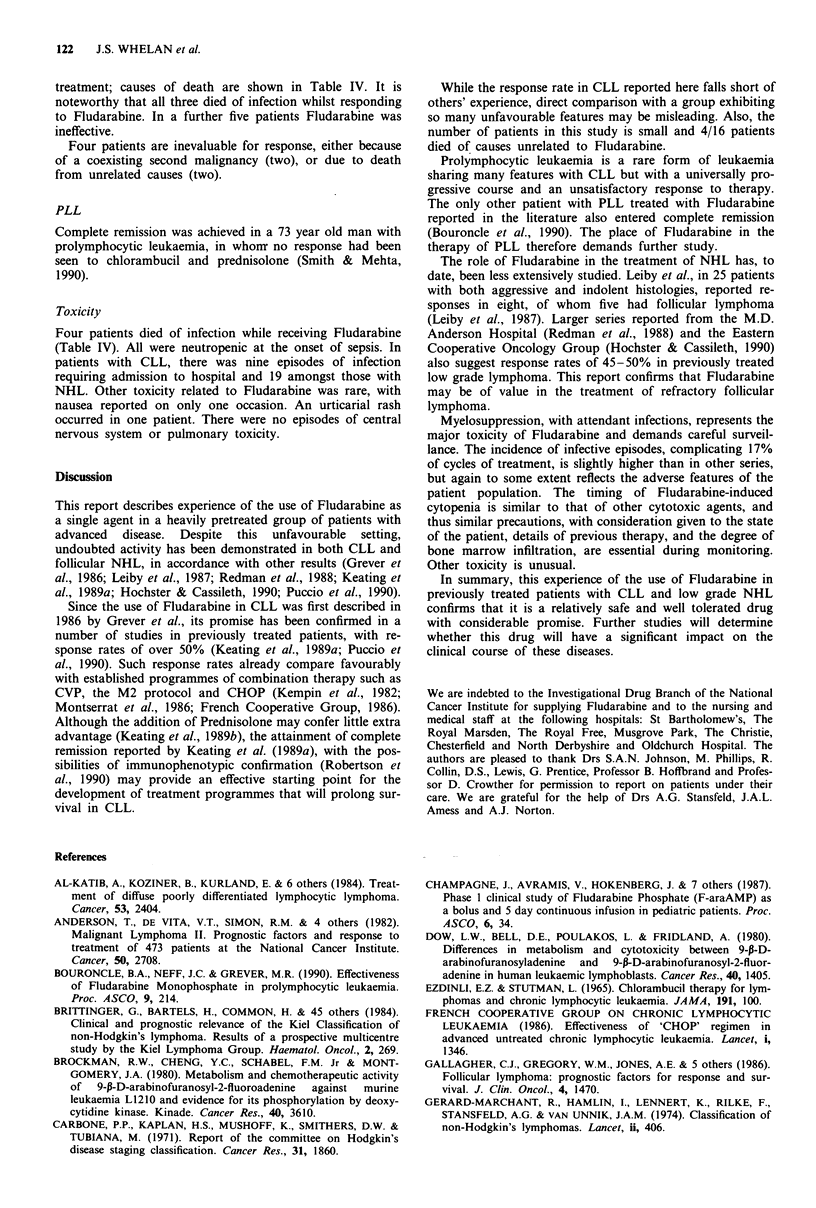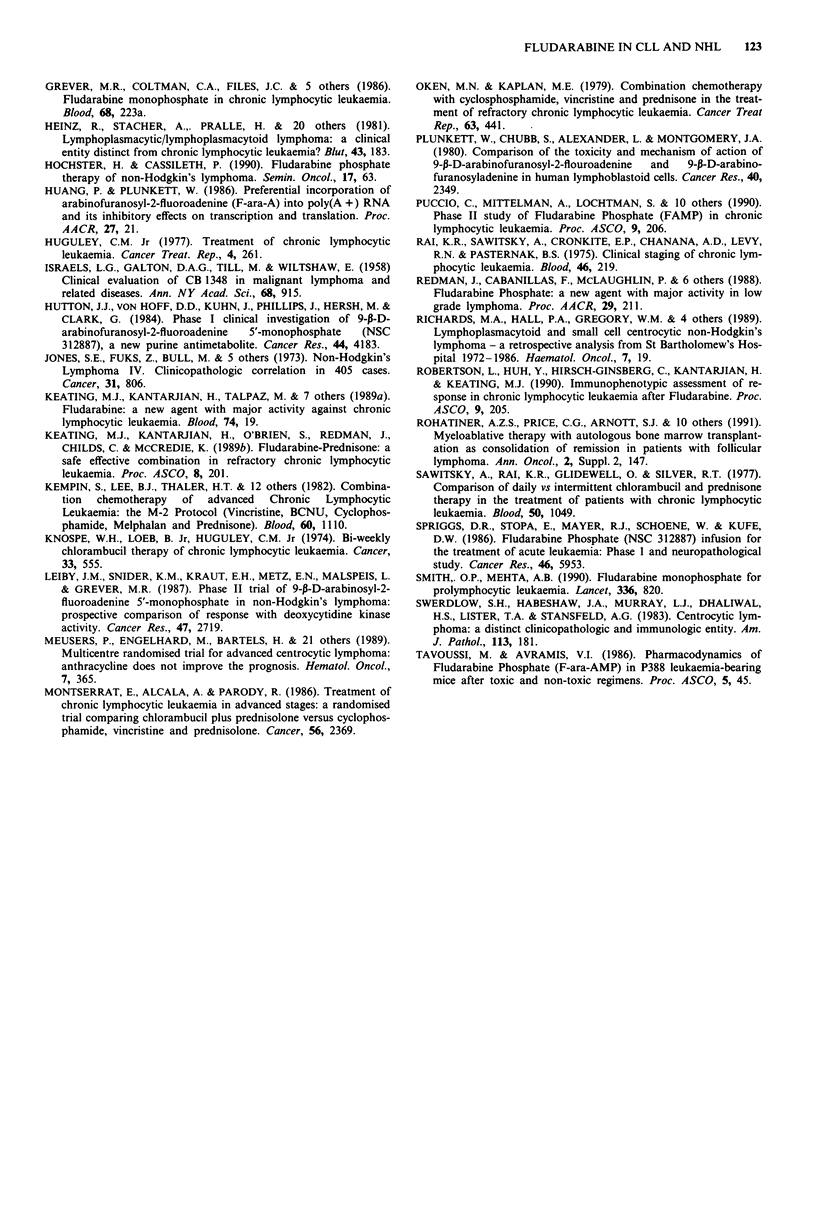# Fludarabine phosphate for the treatment of low grade lymphoid malignancy.

**DOI:** 10.1038/bjc.1991.253

**Published:** 1991-07

**Authors:** J. S. Whelan, C. L. Davis, S. Rule, M. Ranson, O. P. Smith, A. B. Mehta, D. Catovsky, A. Z. Rohatiner, T. A. Lister

**Affiliations:** ICRF Department of Medical Oncology, St Bartholomew's Hospital, London, UK.

## Abstract

Thirty-four patients with previously treated, advanced, low grade NHL were treated with Fludarabine, a deamination-resistant analogue of adenosine arabinoside, at a dose of 25 mg m-2 intravenously, daily for 5 days (median number of cycles = 3, range 1-10). Complete remission (CR) was achieved in six and partial remission (PR) in a further seven. Overall, responses were seen in 11/23 patients (48%) with follicular lymphoma and in 2/11 (18%) with low grade, diffuse NHL. Fifteen patients with previously treated CLL and one patient with prolymphocytic leukaemia (PLL) were also treated as above (median no. of cycles = 3, range 1-6). A partial response was seen in three of the 11 evaluable patients with CLL and CR was achieved in the patient with PLL. There were four deaths due to infection and 19 further episodes requiring admission to hospital. No other significant toxicity was reported in a total of 164 cycles of Fludarabine. This agent is active in advanced low grade lymphoid malignancy. Further studies are required to assess its role in newly diagnosed patients.


Br. J. Cancer (1991), 64, 120-123 © Macmillan Press Ltd., 1991

### Fludarabine phosphate for the treatment of low grade lymphoid malignancy

J.S. Whelan[1], C.L. Davis[1], S. Rule[2,*], M. Ranson[3], O.P. Smith[4], A.B. Mehta[4], D. Catovsky[5], A.Z.S. Rohatiner[1] & T.A. Lister[1]

[1]*ICRF Department of Medical Oncology, St Bartholomew's Hospital, London;* [2]*Musgrove Park Hospital, Taunton, Somerset;* [3]*Christie Hospital, Manchester;* [4]*The Royal Free Hospital, London and* [5]*The Royal Marsden Hospital, London, UK.*

**Summary** Thirty-four patients with previously treated, advanced, low grade NHL were treated with Fludarabine, a deamination-resistant analogue of adenosine arabinoside, at a dose of 25 mg m$^{-2}$ intravenously, daily for 5 days (median number of cycles = 3, range 1-10). Complete remission (CR) was achieved in six and partial remission (PR) in a further seven. Overall, responses were seen in 11/23 patients (48%) with follicular lyphoma and in 2/11 (18%) with low grade, diffuse NHL. Fifteen patients with previously treated CLL and one patient with prolymphocytic leukaemia (PLL) were also treated as above (median no. of cycles = 3, range 1-6). A partial response was seen in three of the 11 evaluable patients with CLL and CR was achieved in the patient with PLL. There were four deaths due to infection and 19 further episodes requiring admission to hospital. No other significant toxicity was reported in a total of 164 cycles of Fludarabine. This agent is active in advanced low grade lymphoid malignancy. Further studies are required to assess its role in newly diagnosed patients.

The low grade lymphoid malignancies are characterised by responsiveness to single agent chemotherapy and by the virtual inevitability of recurrence. Thereafter, although repeated responses may be achieved, the majority of patients die as a consequence of the disease. The alkylating agents, chlorambucil and cyclophosphamide, when used alone, may induce response rates of up to 60% in previously untreated patients with CLL (Knospe *et al.*, 1974; Huguley, 1977; Sawitsky *et al.*, 1977) but lower response rates with a shorter duration of remission are the rule for subsequent therapy (Oken & Kaplan, 1979; Montserrat *et al.*, 1986). Similarly, in follicular NHL, response rates to single agent chemotherapy at presentation and at first and second recurrence remain about 65-70% (Israels *et al.*, 1958; Ezdinli & Stutzman, 1965; Jones *et al.*, 1973; Anderson *et al.*, 1982) but, thereafter, therapy becomes rapidly less satisfactory (Gallagher *et al.*, 1986).

The Kiel classification (Gerard-Marchant *et al.*, 1974) characterises certain diffuse lymphomas as 'low grade' (lymphoplasmacytoid, small cell centrocytic and diffuse centroblastic/centrocytic). The majority of patients present with advanced disease; many respond to alkylating agents, but responses are rarely complete or durable. Hence, fewer than 20% of those with stage III and IV disease survive for more than 5 years (Heinz *et al.*, 1981; Swerdlow *et al.*, 1983; Brittinger *et al.*, 1984; Richards *et al.*, 1989). While responsiveness to chlorambucil falls short of that seen in follicular NHL, the benefits of more intensive therapy in centrocytic lymphoma have yet to be convincingly demonstrated (Al-Katib *et al.*, 1984; Meusers *et al.*, 1989). Better therapy is urgently required.

Fludarabine is a fluorinated analogue of adenosine arabinoside (Ara-A), which retains cytotoxicity while being resistant in the rapid deamination and consequent inactivation which characterises the metabolism of Ara-A (Dow *et al.*, 1980; Plunkett *et al.*, 1980; Brockman *et al.*, 1980; Tavoussi & Avramis, 1986). An active triphosphate metabolite is formed intracellularly which intereferes with DNA synthesis by inhibition of DNA polymerase and ribonucleotide reductase (Huang & Plunkett, 1986). Promise of antitumour activity in murine models has been borne out in Phase I and II testing (Hutton *et al.*, 1984; Spriggs *et al.*, 1986; Champagne *et al.*, 1987). At a dose of $\leqslant$125 mg/m$^2$/course, significant activity, with response rates of 60% and more, has been reported in CLL and follicular NHL, with some modest benefit in diffuse low grade lymphoma (Grever *et al.*, 1986; Leiby *et al.*, 1987; Redman *et al.*, 1988' Hochster & Cassileth, 1990; Keating *et al.*, 1989*a*; Puccio *et al.*, 1990). Fludarabine is reported to be well tolerated at such doses, the toxicity in these studies being predominantly infective episodes arising from myelosuppresion.

This report describes further experience of the efficacy and toxicity of Fludarabine in 50 patients with previously treated low grade lymphoid malignancy.

### Patients and methods

### *Patients*

Thirty four patients with NHL, 15 with CLL, and one with prolymphocytic leukaemia (PLL) form the basis of this study. All had received previous chemotherapy. Their clinical characteristics at the time of treatment with Fludarabine are shown in Tables I and II. For those with NHL, the Kiel Classifiction (Gerard-Marchant *et al.*, 1974), and a modification of the Ann Arbor staging system (Carbone *et al.*, 1971) were used.

**Table I** Characteristics of patients with CLL[a] when treated with Fludarabine

| | | |
|---|---|---|
| Men:Women | 10:6 | |
| Median age | 61 years | (range 43-81) |
| Rai stage 2 | 1 | |
| 3 | 4 | |
| 4 | 11 | |
| Median duration of disease | 75 months | (6-132) |
| Median no. of prior regimens | 3 | (1-8) |
| No. previously treated with: | | |
| anthracyclines | 8 | |
| splenectomy | 3 | |
| splenic irradiation | 5[b] | |
| Mean haemoglobin (g dl)$^{-1}$ | 10.5 | (7.6-13.8) |
| white cell count (10$^9$ l)$^{-1}$ | 159.5 | (6-416) |
| platelet count (10$^9$ l)$^{-1}$ | 109 | (7-355) |

[a]Including one patient with prolymphocytic leukaemia. [b]One patient treated on two occasions.

Correspondence: J. Whelan, ICRF Department of Medical Oncology, St Bartholomew's Hospital, West Smithfield, London EC1A 7BE, UK.
*Current address: Department of Haematology, Westminster Hospital, London, UK.
Received 14 December 1990; and in revised form 31 January 1991.

**Table II** Characteristics of patients with NHL when treated with Fludarabine

| | | | |
|---|---|---|---|
| Men:Women | 18:16 | | |
| Median age | 54 | years | (range 32–73) |
| Histology:[a] Follicular | 23 | | |
| Lymphoplasmacytoid | 4 | | |
| Centrocytic, small cell | 3 | | |
| Lymphocytic | 2 | | |
| Low grade, unclassified | 2 | | |
| Median duration of disease | 47 | months | (4–108) |
| Stage III | 7 | | |
| IV | 27 | | |
| Sites of extranodal disease | | | |
| Bone marrow | 24 | | |
| Peripheral blood | 5 | | |
| Liver | 8 | | |
| Lung | 4 | | |
| Other | 3 | | |
| Median no. of prior regimens | 3 | | (1–7) |
| No. previously treated with: | | | |
| Anthracyclines | 28 | | |
| Splenectomy | 4 | | |

[a]Kiel classification.

*Response criteria*

*NHL* CR was recorded when there was no clinical or radiological evidence of disease and PR when an estimated reduction of at least 50% of tumour volume had occurred. Any lesser response was deemed a treatment failure.

*CLL and PLL* Complete remission was recorded if the peripheral blood lymphocyte count was $<4.0 \times 10^9\,l^{-1}$, bone marrow examination showed $<30\%$ lymphocytes and there were no lymph nodes $>0.5$ cm or hepatosplenomegaly (Rai *et al.*, 1975). Partial remission (PR) was recorded if a $>50\%$ reduction occurred at all sites of disease i.e. circulating lymphocytosis, the degree of bone marrow infiltration, lymphadenopathy and hepatosplenomegaly. Again, a lesser response was deemed a failure of therapy.

*Treatment*

Fludarabine was reconstituted in 10 ml of sterile water and given as a slow intravenous injection. Twenty five mg $m^{-2}$ were given daily for 5 days and repeated every 21–28 days as dictated by the peripheral blood count and clinical circumstances. In one patient, a dose reduction of 50% was made for the first two of six cycles because of an initial low performance status.

Thirty four patients with NHL received 115 cycles of Fludarabine (mean 3, range 1–10) and 49 cycles were given to 15 patients with CLL and one patient with PLL (mean 3, range 1–6). In general, two cycles of therapy were given beyond the best clinically evaluable response. If no response was seen after two cycles of therapy, no further Fludarabine was given. Full supportive therapy with antibiotics and blood products was given as appropriate.

### Results

*NHL*

Response and remission rates according to histology are shown for the 34 patients in Table III.

The response rate (CR + PR) in follicular lymphoma was 11/23 (48%) including five patients in whom CR was achieved. The likelihood of response did not correlate with the number of previous treatments, prior exposure to anthracyclines (21/23 patients), or treatment of recurrent rather than refractory disease.

Two responses were seen in the 11 patients with diffuse histology, one of whom, a 64 year old man with a 5 year history of stage IV lymphoplasmacytoid lymphoma, entered CR. Two patients died of progressive lymphoma and another of a presumed pulmonary embolus. A fourth patient died of infection (Table IV).

No response was seen in 17 patients, including three of four patients who received two cycles of Fludarabine as treatment for 'minimal residual' follicular lymphoma (<10% bone marrow infiltration (two patients, <2 cm lymphadenopathy (one patient)) as the only sites of disease after conventional therapy. Subsequently, these patients received cyclophosphamide and total body irradiation supported by autologous bone marrow transplantation (Rohatiner *et al.*, 1991).

*CLL*

Eleven patients are evaluable for response. No complete remissions were seen. A partial response was observed in three patients (27%). Three patients died whilst receiving

**Table III** Response rates by NHL histology (%)

| | *Follicular* | *Lymphopl'd* | *Centrocytic* | *Lymphocytic* | *Unclass* |
|---|---|---|---|---|---|
| CR | 5 (22) | 1 (25) | – | – | – |
| PR | 6 (26) | – | – | 1 (50) | – |
| Fail | 10 (43) | 3 (75) | 1 (33) | 1 (50) | 2 (100) |
| Death | 2 (9) | – | 2 (66) | – | – |
| Total | 23 | 4 | 3 | 2 | 2 |

**Table IV** Details of patients dying during therapy with Fludarabine

| *Age* | *Diagnosis* | *Duration (months)* | *Stage* | *No. of Prior regimens* | *Karnofky score* | *No. of Flu. cycles* | *Cause of death* | *Response?* |
|---|---|---|---|---|---|---|---|---|
| 60 | NHL | 30 | 3 | 4 | 80% | 2 | Fungal septicaemia | Yes |
| 73 | NHL | 68 | 4 | 4 | 70% | 1 | Pulmonary embolus? | No |
| 56 | NHL | 84 | 4 | 5 | 80% | 2 | NHL | No |
| 46 | NHL | 92 | 4 | 4 | 60% | 1 | Presumed NHL | No |
| 57 | CLL | 47 | 4 | 4[a] | 70% | 2 | Pneumonococcal sepsis | Yes |
| 64 | CLL | 121 | 4 | 6 | 80% | 2 | Pulmonary infection | Yes |
| 68 | CLL | 87 | 4 | 4[b] | 40% | 1 | Septicaemia | Yes |

[a]Including splenectomy. [b]Not including splenic irradiation × 2.

treatment; causes of death are shown in Table IV. It is noteworthy that all three died of infection whilst responding to Fludarabine. In a further five patients Fludarabine was ineffective.

Four patients are inevaluable for response, either because of a coexisting second malignancy (two), or due to death from unrelated causes (two).

### *PLL*

Complete remission was achieved in a 73 year old man with prolymphocytic leukaemia, in whom no response had been seen to chlorambucil and prednisolone (Smith & Mehta, 1990).

### *Toxicity*

Four patients died of infection while receiving Fludarabine (Table IV). All were neutropenic at the onset of sepsis. In patients with CLL, there was nine episodes of infection requiring admission to hospital and 19 amongst those with NHL. Other toxicity related to Fludarabine was rare, with nausea reported on only one occasion. An urticarial rash occurred in one patient. There were no episodes of central nervous system or pulmonary toxicity.

### Discussion

This report describes experience of the use of Fludarabine as a single agent in a heavily pretreated group of patients with advanced disease. Despite this unfavourable setting, undoubted activity has been demonstrated in both CLL and follicular NHL, in accordance with other results (Grever *et al.*, 1986; Leiby *et al.*, 1987; Redman *et al.*, 1988; Keating *et al.*, 1989*a*; Hochster & Cassileth, 1990; Puccio *et al.*, 1990).

Since the use of Fludarabine in CLL was first described in 1986 by Grever *et al.*, its promise has been confirmed in a number of studies in previously treated patients, with response rates of over 50% (Keating *et al.*, 1989*a*; Puccio *et al.*, 1990). Such response rates already compare favourably with established programmes of combination therapy such as CVP, the M2 protocol and CHOP (Kempin *et al.*, 1982; Montserrat *et al.*, 1986; French Cooperative Group, 1986). Although the addition of Prednisolone may confer little extra advantage (Keating *et al.*, 1989*b*), the attainment of complete remission reported by Keating *et al.* (1989*a*), with the possibilities of immunophenotypic confirmation (Robertson *et al.*, 1990) may provide an effective starting point for the development of treatment programmes that will prolong survival in CLL.

While the response rate in CLL reported here falls short of others' experience, direct comparison with a group exhibiting so many unfavourable features may be misleading. Also, the number of patients in this study is small and 4/16 patients died of causes unrelated to Fludarabine.

Prolymphocytic leukaemia is a rare form of leukaemia sharing many features with CLL but with a universally progressive course and an unsatisfactory response to therapy. The only other patient with PLL treated with Fludarabine reported in the literature also entered complete remission (Bouroncle *et al.*, 1990). The place of Fludarabine in the therapy of PLL therefore demands further study.

The role of Fludarabine in the treatment of NHL has, to date, been less extensively studied. Leiby *et al.*, in 25 patients with both aggressive and indolent histologies, reported responses in eight, of whom five had follicular lymphoma (Leiby *et al.*, 1987). Larger series reported from the M.D. Anderson Hospital (Redman *et al.*, 1988) and the Eastern Cooperative Oncology Group (Hochster & Cassileth, 1990) also suggest response rates of 45–50% in previously treated low grade lymphoma. This report confirms that Fludarabine may be of value in the treatment of refractory follicular lymphoma.

Myelosuppression, with attendant infections, represents the major toxicity of Fludarabine and demands careful surveillance. The incidence of infective episodes, complicating 17% of cycles of treatment, is slightly higher than in other series, but again to some extent reflects the adverse features of the patient population. The timing of Fludarabine-induced cytopenia is similar to that of other cytotoxic agents, and thus similar precautions, with consideration given to the state of the patient, details of previous therapy, and the degree of bone marrow infiltration, are essential during monitoring. Other toxicity is unusual.

In summary, this experience of the use of Fludarabine in previously treated patients with CLL and low grade NHL confirms that it is a relatively safe and well tolerated drug with considerable promise. Further studies will determine whether this drug will have a significant impact on the clinical course of these diseases.

We are indebted to the Investigational Drug Branch of the National Cancer Institute for supplying Fludarabine and to the nursing and medical staff at the following hospitals: St Bartholomew's, The Royal Marsden, The Royal Free, Musgrove Park, The Christie, Chesterfield and North Derbyshire and Oldchurch Hospital. The authors are pleased to thank Drs S.A.N. Johnson, M. Phillips, R. Collin, D.S., Lewis, G. Prentice, Professor B. Hoffbrand and Professor D. Crowther for permission to report on patients under their care. We are grateful for the help of Drs A.G. Stansfeld, J.A.L. Amess and A.J. Norton.

### References

AL-KATIB, A., KOZINER, B., KURLAND, E. & 6 others (1984). Treatment of diffuse poorly differentiated lymphocytic lymphoma. *Cancer*, **53**, 2404.

ANDERSON, T., DE VITA, V.T., SIMON, R.M. & 4 others (1982). Malignant Lymphoma II. Prognostic factors and response to treatment of 473 patients at the National Cancer Institute. *Cancer*, **50**, 2708.

BOURONCLE, B.A., NEFF, J.C. & GREVER, M.R. (1990). Effectiveness of Fludarabine Monophosphate in prolymphocytic leukaemia. *Proc. ASCO*, **9**, 214.

BRITTINGER, G., BARTELS, H., COMMON, H. & 45 others (1984). Clinical and prognostic relevance of the Kiel Classification of non-Hodgkin's lymphoma. Results of a prospective multicentre study by the Kiel Lymphoma Group. *Haematol. Oncol.*, **2**, 269.

BROCKMAN, R.W., CHENG, Y.C., SCHABEL, F.M. Jr & MONTGOMERY, J.A. (1980). Metabolism and chemotherapeutic activity of 9-β-D-arabinofuranosyl-2-fluoroadenine against murine leukaemia L1210 and evidence for its phosphorylation by deoxycytidine kinase. Kinade. *Cancer Res.*, **40**, 3610.

CARBONE, P.P., KAPLAN, H.S., MUSHOFF, K., SMITHERS, D.W. & TUBIANA, M. (1971). Report of the committee on Hodgkin's disease staging classification. *Cancer Res.*, **31**, 1860.

CHAMPAGNE, J., AVRAMIS, V., HOKENBERG, J. & 7 others (1987). Phase 1 clinical study of Fludarabine Phosphate (F-araAMP) as a bolus and 5 day continuous infusion in pediatric patients. *Proc. ASCO*, **6**, 34.

DOW, L.W., BELL, D.E., POULAKOS, L. & FRIDLAND, A. (1980). Differences in metabolism and cytotoxicity between 9-β-D-arabinofuranosyladenine and 9-β-D-arabinofuranosyl-2-fluoradenine in human leukaemic lymphoblasts. *Cancer Res.*, **40**, 1405.

EZDINLI, E.Z. & STUTMAN, L. (1965). Chlorambucil therapy for lymphomas and chronic lymphocytic leukaemia. *JAMA*, **191**, 100.

FRENCH COOPERATIVE GROUP ON CHRONIC LYMPHOCYTIC LEUKAEMIA (1986). Effectiveness of 'CHOP' regimen in advanced untreated chronic lymphocytic leukaemia. *Lancet*, **i**, 1346.

GALLAGHER, C.J., GREGORY, W.M., JONES, A.E. & 5 others (1986). Follicular lymphoma: prognostic factors for response and survival. *J. Clin. Oncol.*, **4**, 1470.

GERARD-MARCHANT, R., HAMLIN, I., LENNERT, K., RILKE, F., STANSFELD, A.G. & VAN UNNIK, J.A.M. (1974). Classification of non-Hodgkin's lymphomas. *Lancet*, **ii**, 406.

GREVER, M.R., COLTMAN, C.A., FILES, J.C. & 5 others (1986). Fludarabine monophosphate in chronic lymphocytic leukaemia. *Blood*, **68**, 223a.

HEINZ, R., STACHER, A.,. PRALLE, H. & 20 others (1981). Lymphoplasmacytic/lymphoplasmacytoid lymphoma: a clinical entity distinct from chronic lymphocytic leukaemia? *Blut*, **43**, 183.

HOCHSTER, H. & CASSILETH, P. (1990). Fludarabine phosphate therapy of non-Hodgkin's lymphoma. *Semin. Oncol.*, **17**, 63.

HUANG, P. & PLUNKETT, W. (1986). Preferential incorporation of arabinofuranosyl-2-fluoroadenine (F-ara-A) into poly(A +) RNA and its inhibitory effects on transcription and translation. *Proc. AACR*, **27**, 21.

HUGULEY, C.M. Jr (1977). Treatment of chronic lymphocytic leukaemia. *Cancer Treat. Rep.*, **4**, 261.

ISRAELS, L.G., GALTON, D.A.G., TILL, M. & WILTSHAW, E. (1958) Clinical evaluation of CB 1348 in malignant lymphoma and related diseases. *Ann. NY Acad. Sci.*, **68**, 915.

HUTTON, J.J., VON HOFF, D.D., KUHN, J., PHILLIPS, J., HERSH, M. & CLARK, G. (1984). Phase I clinical investigation of 9-β-D-arabinofuranosyl-2-fluoroadenine 5'-monophosphate (NSC 312887), a new purine antimetabolite. *Cancer Res.*, **44**, 4183.

JONES, S.E., FUKS, Z., BULL, M. & 5 others (1973). Non-Hodgkin's Lymphoma IV. Clinicopathologic correlation in 405 cases. *Cancer*, **31**, 806.

KEATING, M.J., KANTARJIAN, H., TALPAZ, M. & 7 others (1989*a*). Fludarabine: a new agent with major activity against chronic lymphocytic leukaemia. *Blood*, **74**, 19.

KEATING, M.J., KANTARJIAN, H., O'BRIEN, S., REDMAN, J., CHILDS, C. & MCCREDIE, K. (1989*b*). Fludarabine-Prednisone: a safe effective combination in refractory chronic lymphocytic leukaemia. *Proc. ASCO*, **8**, 201.

KEMPIN, S., LEE, B.J., THALER, H.T. & 12 others (1982). Combination chemotherapy of advanced Chronic Lymphocytic Leukaemia: the M-2 Protocol (Vincristine, BCNU, Cyclophosphamide, Melphalan and Prednisone). *Blood*, **60**, 1110.

KNOSPE, W.H., LOEB, B. Jr, HUGULEY, C.M. Jr (1974). Bi-weekly chlorambucil therapy of chronic lymphocytic leukaemia. *Cancer*, **33**, 555.

LEIBY, J.M., SNIDER, K.M., KRAUT, E.H., METZ, E.N., MALSPEIS, L. & GREVER, M.R. (1987). Phase II trial of 9-β-D-arabinosyl-2-fluoroadenine 5'-monophosphate in non-Hodgkin's lymphoma: prospective comparison of response with deoxycytidine kinase activity. *Cancer Res.*, **47**, 2719.

MEUSERS, P., ENGELHARD, M., BARTELS, H. & 21 others (1989). Multicentre randomised trial for advanced centrocytic lymphoma: anthracycline does not improve the prognosis. *Hematol. Oncol.*, **7**, 365.

MONTSERRAT, E., ALCALA, A. & PARODY, R. (1986). Treatment of chronic lymphocytic leukaemia in advanced stages: a randomised trial comparing chlorambucil plus prednisolone versus cyclophosphamide, vincristine and prednisolone. *Cancer*, **56**, 2369.

OKEN, M.N. & KAPLAN, M.E. (1979). Combination chemotherapy with cyclosphosphamide, vincristine and prednisone in the treatment of refractory chronic lymphocytic leukaemia. *Cancer Treat Rep.*, **63**, 441.

PLUNKETT, W., CHUBB, S., ALEXANDER, L. & MONTGOMERY, J.A. (1980). Comparison of the toxicity and mechanism of action of 9-β-D-arabinofuranosyl-2-flouroadenine and 9-β-D-arabinofuranosyladenine in human lymphoblastoid cells. *Cancer Res.*, **40**, 2349.

PUCCIO, C., MITTELMAN, A., LOCHTMAN, S. & 10 others (1990). Phase II study of Fludarabine Phosphate (FAMP) in chronic lymphocytic leukaemia. *Proc. ASCO*, **9**, 206.

RAI, K.R., SAWITSKY, A., CRONKITE, E.P., CHANANA, A.D., LEVY, R.N. & PASTERNAK, B.S. (1975). Clinical staging of chronic lymphocytic leukaemia. *Blood*, **46**, 219.

REDMAN, J., CABANILLAS, F., MCLAUGHLIN, P. & 6 others (1988). Fludarabine Phosphate: a new agent with major activity in low grade lymphoma. *Proc. AACR*, **29**, 211.

RICHARDS, M.A., HALL, P.A., GREGORY, W.M. & 4 others (1989). Lymphoplasmacytoid and small cell centrocytic non-Hodgkin's lymphoma – a retrospective analysis from St Bartholomew's Hospital 1972–1986. *Haematol. Oncol.*, **7**, 19.

ROBERTSON, L., HUH, Y., HIRSCH-GINSBERG, C., KANTARJIAN, H. & KEATING, M.J. (1990). Immunophenotypic assessment of response in chronic lymphocytic leukaemia after Fludarabine. *Proc. ASCO*, **9**, 205.

ROHATINER, A.Z.S., PRICE, C.G., ARNOTT, S.J. & 10 others (1991). Myeloablative therapy with autologous bone marrow transplantation as consolidation of remission in patients with follicular lymphoma. *Ann. Oncol.*, **2**, Suppl. 2, 147.

SAWITSKY, A., RAI, K.R., GLIDEWELL, O. & SILVER, R.T. (1977). Comparison of daily *vs* intermittent chlorambucil and prednisone therapy in the treatment of patients with chronic lymphocytic leukaemia. *Blood*, **50**, 1049.

SPRIGGS, D.R., STOPA, E., MAYER, R.J., SCHOENE, W. & KUFE, D.W. (1986). Fludarabine Phosphate (NSC 312887) infusion for the treatment of acute leukaemia: Phase 1 and neuropathological study. *Cancer Res.*, **46**, 5953.

SMITH,. O.P., MEHTA, A.B. (1990). Fludarabine monophosphate for prolymphocytic leukaemia. *Lancet*, **336**, 820.

SWERDLOW, S.H., HABESHAW, J.A., MURRAY, L.J., DHALIWAL, H.S., LISTER, T.A. & STANSFELD, A.G. (1983). Centrocytic lymphoma: a distinct clinicopathologic and immunologic entity. *Am. J. Pathol.*, **113**, 181.

TAVOUSSI, M. & AVRAMIS, V.I. (1986). Pharmacodynamics of Fludarabine Phosphate (F-ara-AMP) in P388 leukaemia-bearing mice after toxic and non-toxic regimens. *Proc. ASCO*, **5**, 45.